# Influence of dynamic load and water on energy accumulation and dissipation in sandstone

**DOI:** 10.1038/s41598-023-49319-3

**Published:** 2023-12-12

**Authors:** Yang Yang, Yulong Xing, Kailun Fang, Chao Wu, Kaiping Yang, Zhifeng Xie, Xianpeng Wang, Leonovich Sergey Nikolayevich

**Affiliations:** 1 China Center for Safety Research, MEM, Beijing, 100013 China; 2 Hebei Haowei Xuguang New Material Technology Co. Ltd., Hebei, 057350 China; 3 Guangzhou Urban Planning and Design Co., Ltd., Guangzhou, 510030 China; 4 State Key Laboratory of Hydroscience and Hydraulic Engineering, Tsinghua University, Beijing, 100084 China; 5 Belarusian National Technical University, Minsk, Belarus

**Keywords:** Solid Earth sciences, Geology

## Abstract

In various engineering projects such as mineral extraction, hydropower resource utilization, railway construction, and geological hazard mitigation, rock engineering is often encountered. Furthermore, dynamic loads and moisture content exert notable influence on the energy transformation processes within rocks. Yet, the specific interplay of dynamic loading and water's impact on the energy conversion mechanism within the sandstone remains unexplored. To address this gap, this study conducted impact loading experiments on sandstone, elucidating the rock’s mechanical response under these conditions and unraveling the underlying energy conversion mechanisms. It was observed that the strength of sandstone exhibits a direct correlation with impact velocity. Moreover, employing energy calculation principles, we established a connection between moisture content and the sandstone’s internal energy conversion properties. The study also delved into the microscopic fracture mechanisms within the sandstone, ultimately concluding that both water content and dynamic loading have a significant impact on these microscopic fracture mechanisms.

## Introduction

In recent years, significant advancements in science and technology have led to the expansion of mining operations to greater depths. Deep mining operations expose rock masses to a complex geological milieu characterized by elevated temperature, pressure, osmotic forces, and disturbances^1–6^. A thorough understanding of the mechanical response of the rock mass in this challenging environment is gained in the support of underground engineering initiatives^7–11^. Nevertheless, the energy conversion mechanisms governing sandstone under the combined effects of water and dynamic loading remain largely uncharted. Hence, there exists a pressing need to investigate and elucidate this intricate energy conversion process.

Numerous domestic and international studies have delved into the effect of water on the rock's mechanical behavior. As an example, Cai examined the rock’s dynamic behavior under high-strain-rate conditions and unveiled substantial alterations in the internal fracture mechanism when water was introduced. Specifically, under dry conditions, the predominant mode of fracture is intragranular rupture, whereas saturated conditions favor intergranular rupture as the primary particle rupture mechanism^12–17^. Reza Khajevand, utilizing neural networks, introduced three distinct models to investigate the uniaxial compressive strength, a pivotal parameter in rock research. These models demonstrated the ability to accurately predict uniaxial compressive strength, thus proposing a novel avenue for non-destructive testing. Additionally, Reza Khajevand developed a predictive model for the durability index of sedimentary rock lakes using experimental data. Simultaneously, mechanical properties of rocks under various conditions were calculated, providing valuable references for rock strength estimation and holding significant importance in rock engineering construction^18–21^.

Li conducted experiments involving sandstone specimens subjected to varying soaking times to achieve different saturation degrees. The aim of the work was to examine how the degree of saturation affects the sandstone mechanical properties when it is compressed uniaxially. The results exhibited that the saturation degree and the sandstone dissipation energy were positively correlated, indicating an increase in dissipation energy with higher saturation degrees. Furthermore, variations in acoustic emission energy evolution were observed corresponding to different saturation degrees^22^.

Zhao conducted a comprehensive study on the influence of various water conditions (dry, natural, and saturated) on the instability and failure of sandstone. The research unveiled significant variations in sandstone strength under different water conditions and highlighted alterations in the rock’s failure mode. Acoustic emission (AE) technology was employed to investigate the influence of water content at each stage of sandstone behavior^23^. Yin conducted a comprehensive investigation utilizing Digital Image Correlation (DIC) and AE methods to investigate how sandstone's mechanical properties are affected by cracks and water content. The results of the investigation displayed that the mechanical characteristics varied significantly based on the water content. Furthermore, along with significant differences in DIC evolution and micro-fracture mechanisms associated with varying fracture lengths^24^. Gu conducted dynamic impact tests on various types of sandstones with differing porosity and water content, establishing a substantial correlation between water saturation and the mechanical behavior of sandstones, particularly concerning variations in porosity. Additionally, a novel mechanical model based on the Stefan effect was developed^25^. Kim thoroughly examined the link between sandstone's compressive and tensile strengths while taking diverse loading rates and levels of water content into account. The research findings demonstrated substantial differences in the tensile and compressive strength across different types of sandstone. Moreover, the study revealed that the sandstone tensile strength exhibited greater sensitivity to variations in loading rate compared to its compressive strength^26^. Zhou performed a series of dynamic tests on sandstone, investigating its behavior under varying saturation conditions. The research findings demonstrated a noteworthy disparity in the fracture rate between dry and saturated rock samples. Moreover, the saturated rock samples exhibited a considerably higher sensitivity to fracture rates compared to their dry counterparts. To provide an explanation for these observations, a corresponding model was developed^13,27–33^. Lu conducted a comprehensive investigation on the sandstone’s dynamic behavior, specifically examining the impact of water content. The study introduced a novel ductility coefficient to assess this effect. Additionally, the research delved into the microscopic fracture mechanism of sandstone through SEM scanning. Notably, the findings revealed an evident association between the ductility coefficient and microscopic observations, demonstrating their mutually consistent nature^34^. Li’s study unveiled a positive correlation between water content and the failure strain of the samples. Furthermore, it identified variations in the crack propagation mode associated with different water content levels. Additionally, the study highlighted distinct microscopic characteristics exhibited by sandstones at varying water content levels^35,36^. Li tested the effects of dynamic loads on sandstone specimens that were both moist and dry. The research findings revealed a significant influence of stress wave energy on the damage degree in the sandstone, while the pore diameter remained unaffected. Furthermore, notable distinctions were observed in the energy conversion characteristics between saturated and dry sandstone^37^. Qi conducted a comprehensive investigation into the effect of water and temperature on the tensile strength of the porous sandstone. The study revealed a strong correlation between temperature and the sandstone dynamic tensile strength. Furthermore, a functional relationship was established between water temperature and the peak strain observed. The utilization of XRD and SEM tests provided conclusive evidence demonstrating that alterations in water temperature have the ability to modify the internal structure of sandstone, consequently affecting its dynamic properties^38^. Zhang tested various impact speeds on sandstone, and the findings suggested that the impact velocity had a major influence on the rock's mechanical characteristics, in addition to proposing some new mechanical coefficients^39^. Liu examined the dynamic tensile properties of saturated and dry sandstone in great detail. The findings demonstrated a notable correlation between prestress and the sandstone dynamic tensile strength. Additionally, the research shed light on the microscopic fracture mechanisms observed in both dry and saturated sandstone^40^. Liu conducted dynamic load analyses on sandstone samples with diverse levels of water saturation and subjected to different microwave radiation. The research findings demonstrated significant variations in the roughness of rock samples associated with different water content. Moreover, the study revealed that different degrees of rock damage occurred due to distinct microwave radiation levels^41^. Wang's research shows that water content is positively correlated with DIF of sandstone^42–44^.

In summary, extensive research has been conducted by scholars both domestically and internationally on the failure properties, deformation as well as strength of the water-bearing rocks. Under the combined impact of water and dynamic stress, the internal energy conversion process of sandstone is yet unknown. Therefore, this study aims to fill this knowledge gap. Three types of sandstone were applied for the Split Hopkinson Pressure Bar (SHPB) tests: natural, dry, and water-saturated. The core of the study is a comprehensive analysis of the mechanical behavior of these sandstones, including strength characteristics, deformation behavior, internal energy evolution, internal energy conversion processes, and energy ratios. The expected results have the potential to provide valuable insights to support underground engineering programs.

## Materials and methods

### Materials

**Figure 1:**
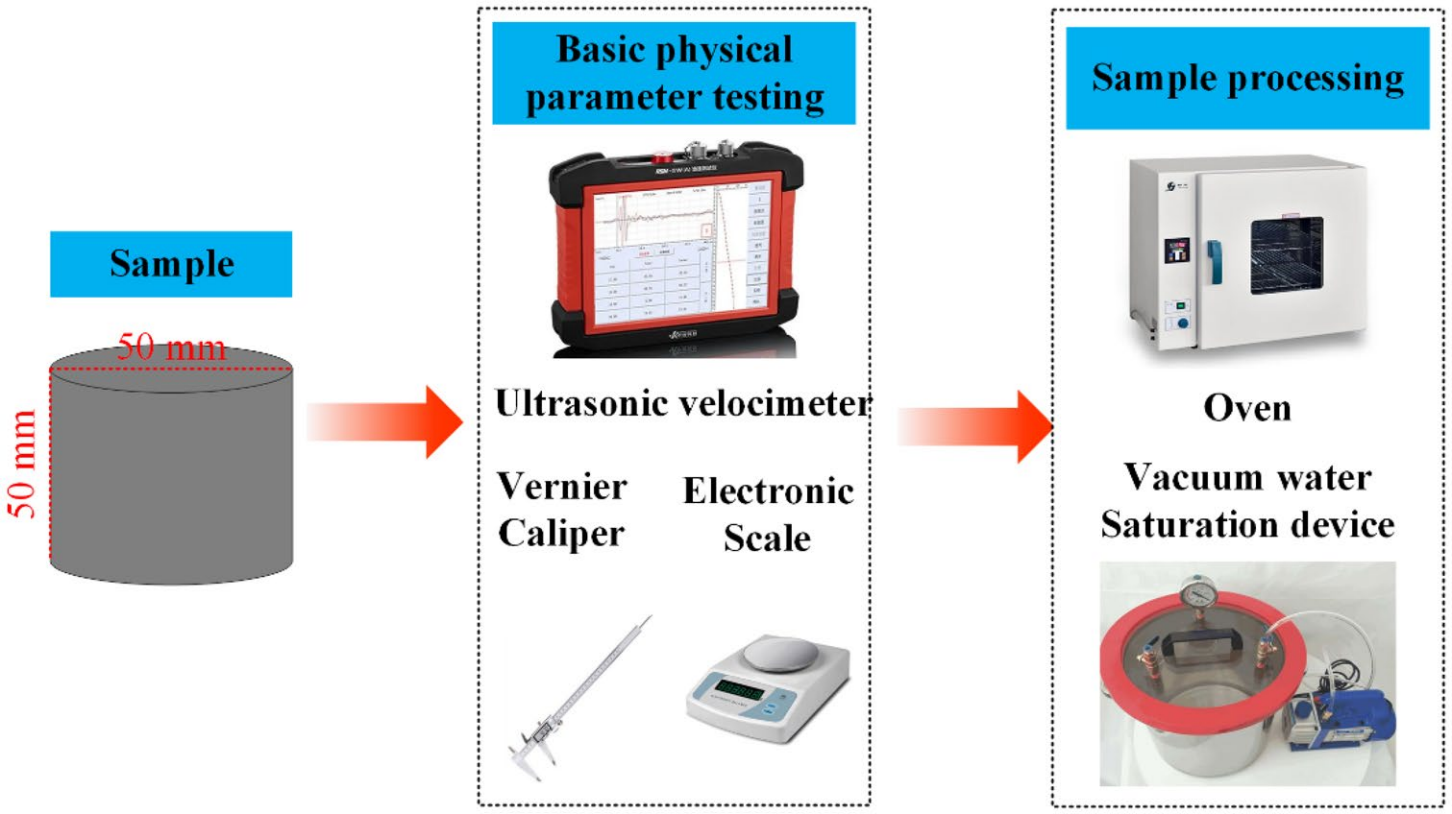
Sample processing.

**Figure 2:**
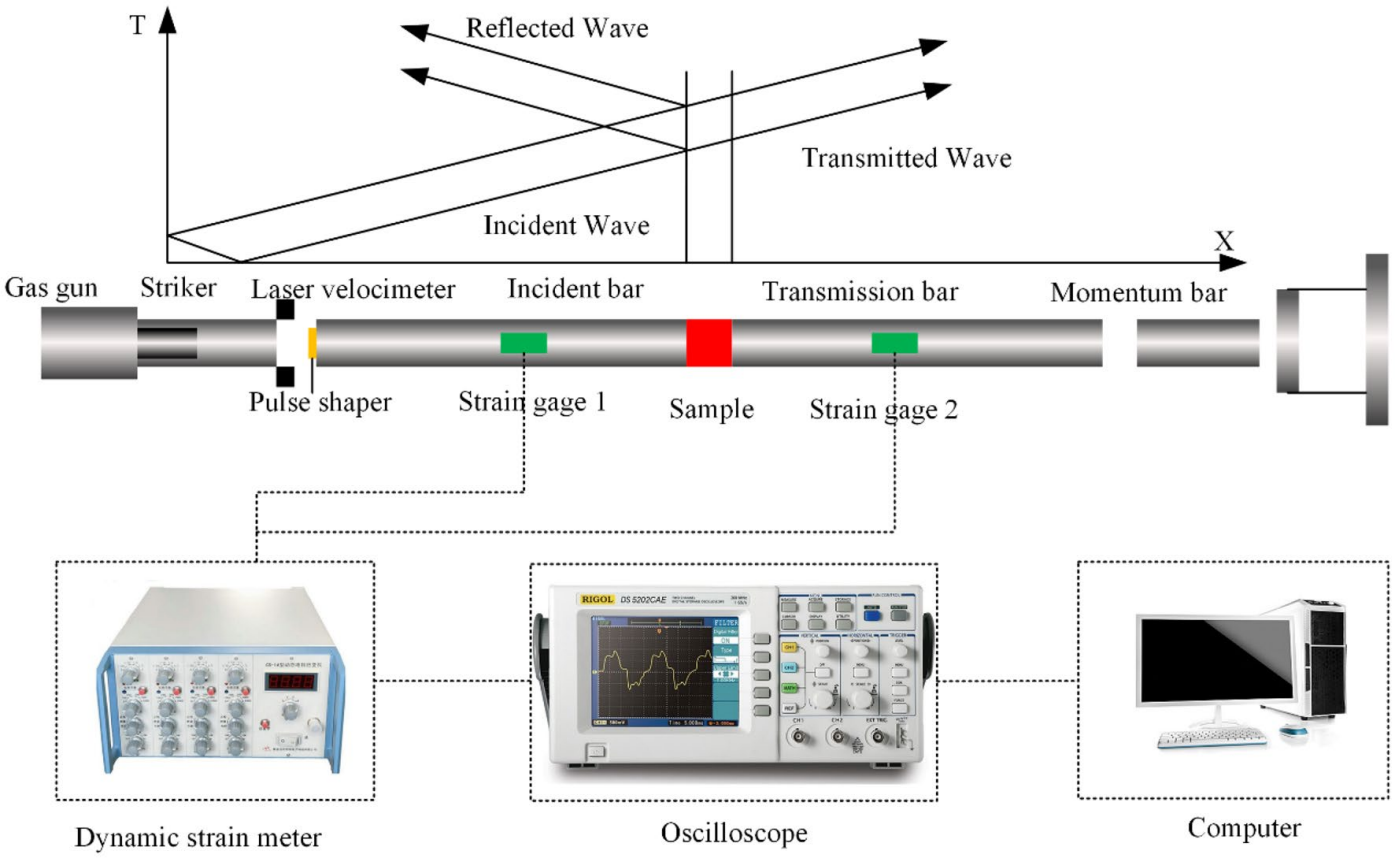
SHPB.

The specimens utilized in this study were sourced from a mine situated in Shanxi Province, China. Shanxi Province is known for its rugged terrain and is intersected by two major river systems, namely, the Yellow River and the Haihe River. The region’s river systems fall under the category of self-producing and outward-flowing types, benefiting from ample rainfall due to its temperate continental monsoon climate. The sandstone specimens selected for the study were meticulously fashioned into cylindrical shapes measuring 50 mm in diameter and 50 mm in height. Figure 1 provides a depiction of the chosen sandstone samples, showcasing their intact surfaces, and highlights that these samples underwent both drying and natural water-filling treatments. Additionally, to ensure adherence to international rock mechanics test standards, the sandstone specimens were prepared accordingly. To facilitate dynamic load impact tests, as illustrated in Fig. 2, the designated natural sandstone, dry sandstone, and saturated sandstone samples underwent testing using their SHPB apparatus.

### Experimental methods

The study initiated with dynamic load impact tests performed on dry sandstone, saturated sandstone, and natural sandstone, utilizing different bullet velocities (4, 8, 12 m/s). The primary objective was to investigate the impact of various dynamic loads on the mechanical properties and internal energy conversion mechanisms within these sandstone variants. The testing procedures employed specific formulas, as referenced by^45,46^, to capture essential data points for instance strain rate, strain, and stress of rock samples.

1 $$\left\{ \begin{gathered} \sigma (t) = \frac{AE}{{A_{0} }}\varepsilon_{{\text{t}}} (t) \hfill \\ \dot{\varepsilon }(t) = \frac{{{\text{c}}_{0} }}{{{\text{l}}_{{\text{s}}} }}[\varepsilon_{{\text{i}}} (t) - \varepsilon_{{\text{r}}} (t) - \varepsilon_{{\text{t}}} (t)] \hfill \\ \varepsilon (t) = \frac{{{\text{c}}_{0} }}{{{\text{l}}_{{\text{s}}} }}\int_{0}^{{\text{t}}} {[\varepsilon_{{\text{i}}} (t) - \varepsilon_{{\text{r}}} (t) - \varepsilon_{{\text{t}}} (t)]} \hfill \\ \end{gathered} \right\}$$

**Figure 3:**
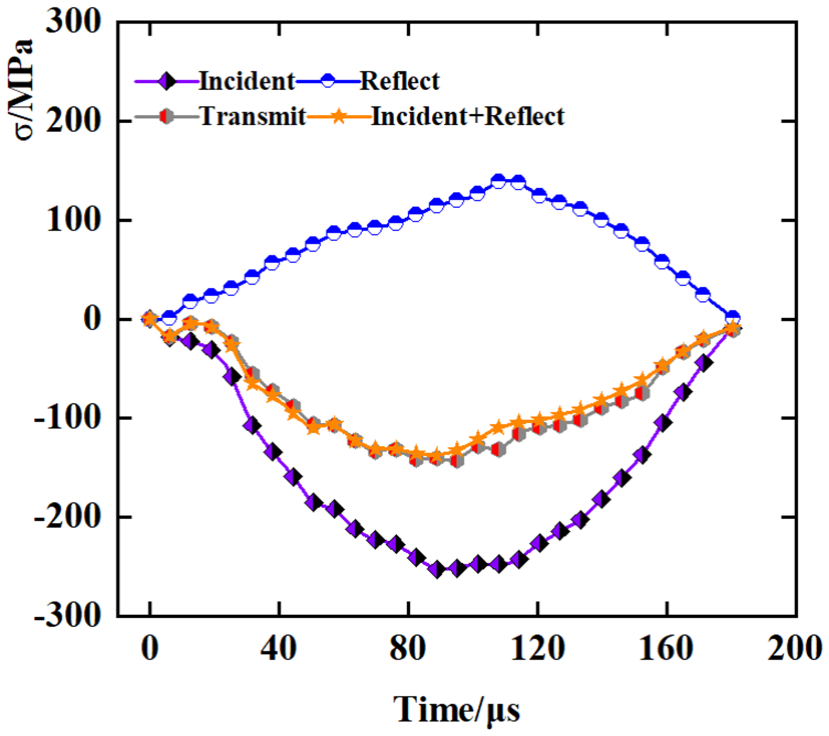
Sample stress balance.

Herein, *A* denotes the sample’s cross-sectional area, and l_s_ stands for its length. Additionally, c_0_ denotes the longitudinal wave velocity, *A*_0_ represents the cross-sectional area, and *E* signifies the elastic modulus of bar body. Moreover, $\varepsilon_{{\text{t}}} (t)$ $\varepsilon_{{\text{i}}} (t)$, and $\varepsilon_{{\text{r}}} (t)$ represent the transmitted, reflected, and incident waves, separately. Figure 3 illustrates that the test conforms to stress equilibrium principles.

## Results

### Stress–strain curve analysis

**Figure 4:**
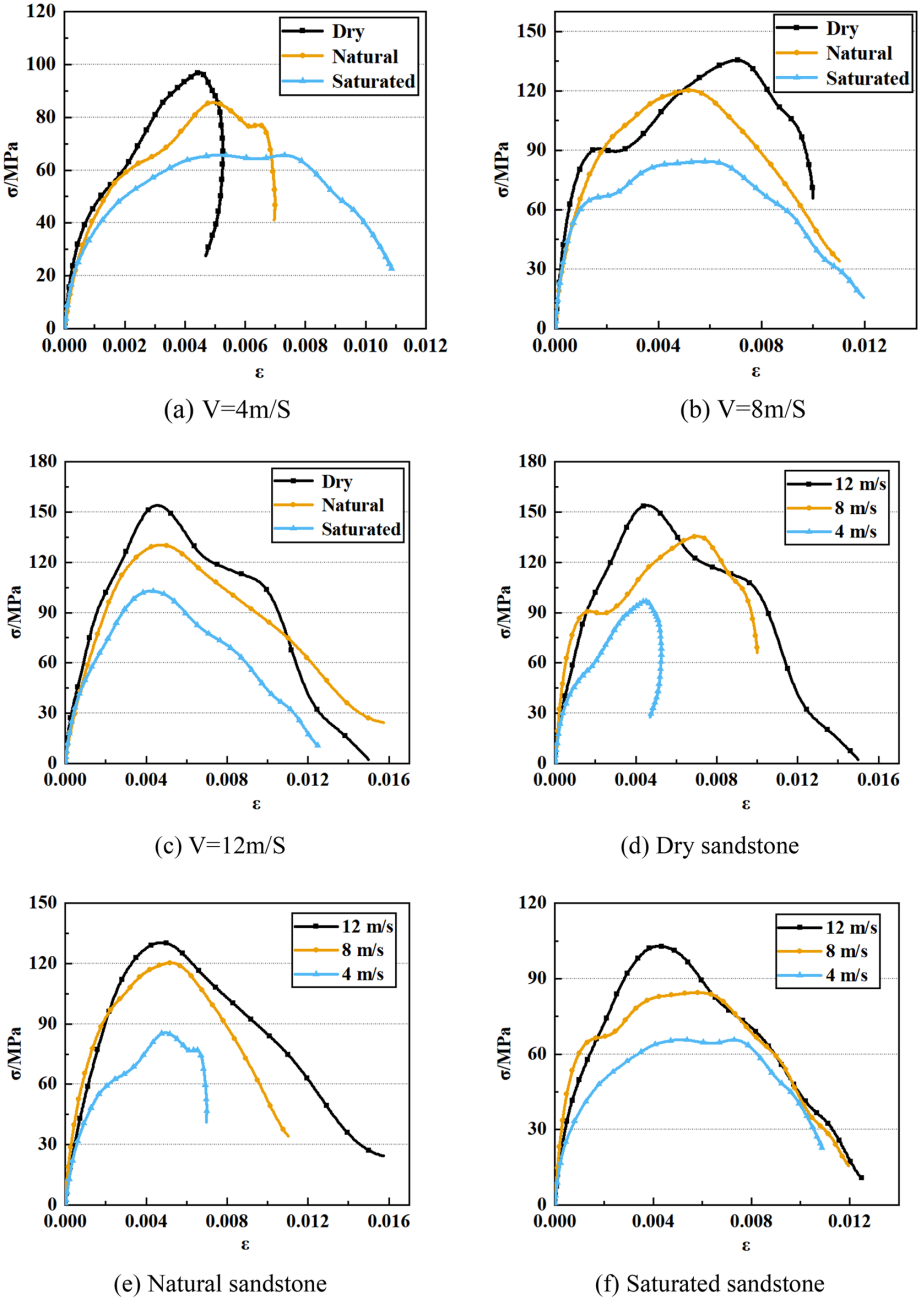
Sandstone’s stress–strain curve.

The stress–strain profiles of natural sandstone, saturated sandstone as well as dry sandstone are presented in Fig. 4. There are four major stages to these curves: compaction, linear elasticity, plasticity, and post-peak behavior. It is evident that both dynamic loading and water saturation exert significant effects on the stress–strain response of sandstone. Notably, with the rise in the impact velocity, the slope of the stress–strain curve in the plasticity phase gradually escalates for dry sandstone, saturated sandstone, and natural sandstone. Furthermore, the peak value for stress–strain curves exhibits an ascending trend with higher impact velocities. It is also noteworthy that the peak value for stress–strain curve is higher for dry sandstone in comparison to both saturated and natural sandstone. These findings underscore the effect of impact velocity and water content on the distinctive characteristics of the stress–strain curve in sandstone.

### Strength characteristics

**Figure 5:**
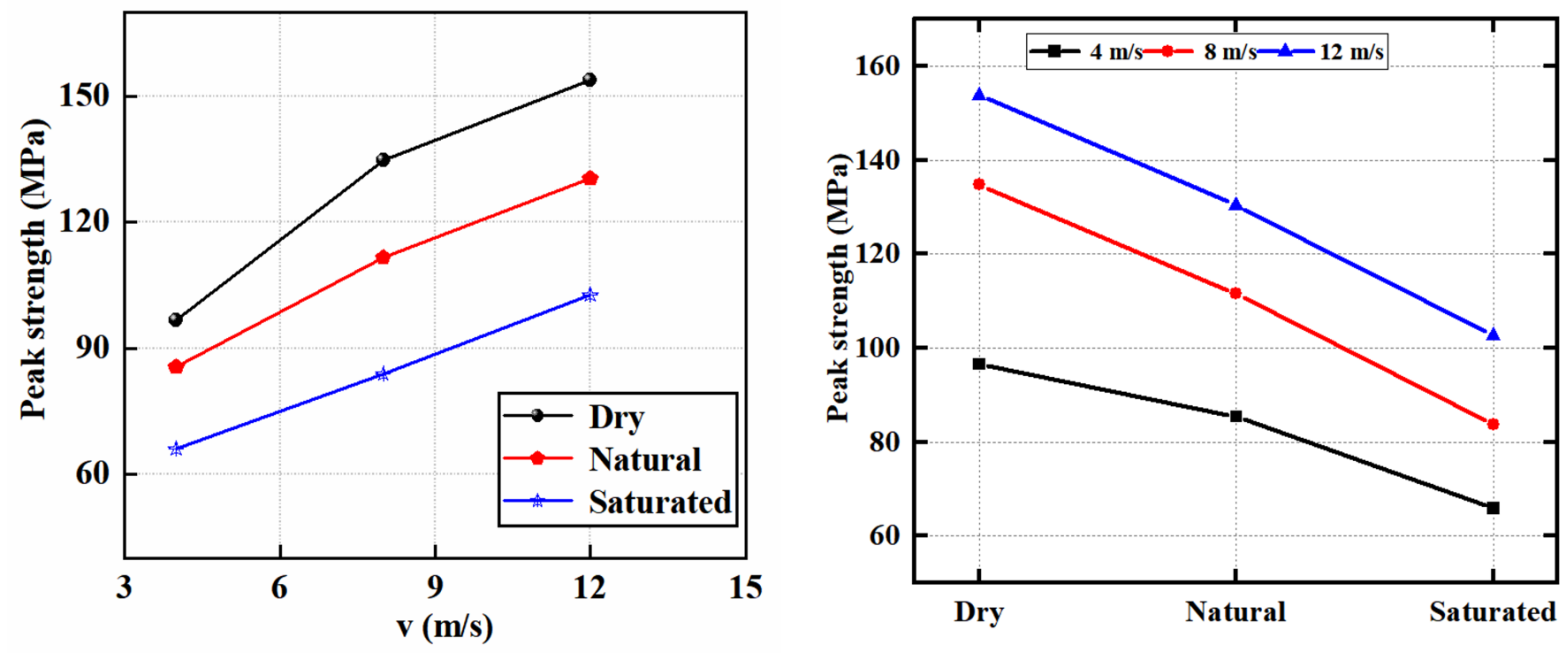
Strength characteristics of sandstone.

Comprehending the evolution of rock mass strength under diverse conditions is paramount to guarantee the effectiveness and safety of rock mass engineering projects. In laboratory settings, the evaluation of rock strength involves conducting loading tests under various conditions. Consequently, this study scrutinizes the strength evolution of dry sandstone, saturated sandstone, and natural sandstone under different dynamic load conditions by conducting dynamic load tests at varying impact velocities. The pertinent insights are presented in Table 1 and Fig. 5, demonstrating the significant impact of saturation level and dynamic stress on the evolution of sandstone strength. As the impact velocity escalates, the strength of dry sandstone, saturated sandstone, and natural sandstone demonstrates an ascending trajectory. Additionally, when compared at identical impact velocities, dry sandstone exhibits superior strength relative to both natural and saturated sandstone counterparts. For instance, at 4 m/s impact velocity, the strength values are recorded as 96.60 MPa, 85.45 MPa, and 65.96 MPa for dry sandstone, saturated sandstone, and natural sandstone, respectively, resulting in a substantial strength disparity of 30.64 MPa between dry and saturated sandstone. Correspondingly, at 8 and 12 m/s impact velocities, the strength differentials between dry and saturated sandstone stand at 50.96 MPa and 51.11 MPa, respectively, with the respective strength values provided for each sandstone type. These findings underscore the pronounced influence of both water content and impact velocity on rock strength properties, with the presence of water leading to a notable attenuation in rock strength.

**Table 1:** Strength characteristics of sandstone.

Bullet velocity (m/s)	Water content	Peak strength (MPa)
4	Dry	96.60
	Natural	85.45
	Saturated	65.96
8	Dry	134.74
	Natural	111.59
	Saturated	83.78
12	Dry	153.81
	Natural	130.40
	Saturated	102.70

### Deformation characteristics

**Figure 6:**
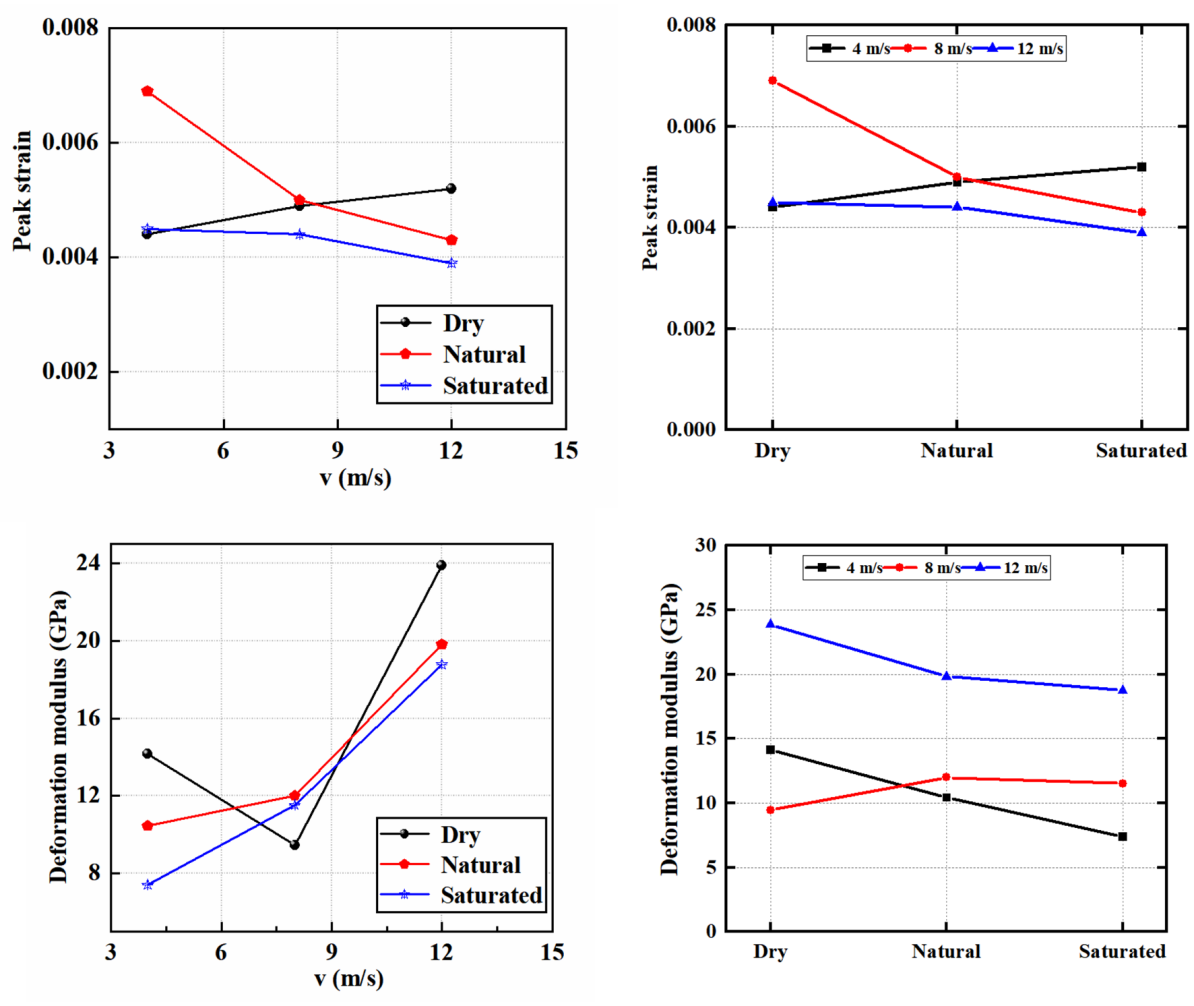
Deformation characteristics of sandstone.

The deformation behavior of rocks holds immense significance in ensuring engineering stability. This study is dedicated to the comprehensive examination of the deformation characteristics exhibited by dry, natural and saturate sandstone when subjected to various dynamic loading conditions through impact testing. The salient insights gleaned from these investigations are aptly represented in Table 2 and Fig. 6, effectively illustrating the profound impact that water saturation and dynamic loading exert on the sandstone deformation characteristics. At lower impact velocities, the sandstone peak strain has a noticeable rising trend as impact velocity increases. In contrast, with greater impact velocities, the peak strain of sandstone demonstrates a decline as impact velocity increases. It is worth noting that, particularly at elevated impact velocities, dry sandstone exhibits peak strain values surpassing those of both natural and saturated sandstone. For instance, at 4 m/s, the peak strain values for dry sandstone, saturated sandstone, and natural sandstone stand at 0.0044, 0.0049, and 0.0052, respectively. Analogously, peak strain values for each sandstone type at 8 m/s and 12 m/s are provided.

**Table 2:** Deformation characteristics of sandstone.

Bullet velocity (m/s)	Water content	Peak strain	Deformation modules (GPa)
4	Dry	0.0044	14.14
	Natural	0.0049	10.44
	Saturated	0.0052	7.39
8	Dry	0.0069	9.46
	Natural	0.0050	11.99
	Saturated	0.0043	11.52
12	Dry	0.0045	23.88
	Natural	0.0044	19.81
	Saturated	0.0039	18.77

The deformation modulus of rock stands as a critical indicator for assessing its deformability. Figure 6 provides a clear visualization of the substantial influence that dynamic loading and water saturation exert on the deformation modulus of sandstone. As the impact velocity escalates, the deformation modulus of dry sandstone, saturated sandstone, and natural sandstone generally exhibits an ascending trajectory. Furthermore, at a given impact velocity, the deformation modulus of dry sandstone surpasses that of both natural and saturated sandstone. This observation underscores the significant impact of water content and impact velocity on the rock deformation characteristics, with the presence of water leading to a reduction in deformation properties. It is noteworthy that an exception arises in the deformation behavior of dry sandstone at 8 m/s impact velocity, which may potentially be attributed to rock dispersion. Consequently, when evaluating rock strength in underground engineering, the effects of both rock moisture content and dynamic loading must be considered.

### Influence mechanism of water-dynamic load on sandstone strength

Building upon insights gleaned from previous research, it becomes evident that the mechanical response of sandstone is profoundly affected by the interplay between dynamic loading and water content. This section is dedicated to a deeper exploration of the fundamental mechanism by which the combined influence of dynamic loading and water impacts the sandstone strength. Rocks, composed of a variety of chemical compounds, undergo extensive chemical reactions when exposed to water. This transformation entails the conversion of high-strength constituents within the rock into lower-strength materials, leading to a discernible reduction in the overall rock strength. To illustrate, consider the case of montmorillonite, a component found in rocks, which undergoes chemical reactions upon contact with water, represented as follows:

$${\text{AI}}_{4} {\text{SI}}_{4} {\text{O}}_{10} ({\text{OH}})_{2} + {\text{nH}}_{2} {\text{O }} \to {\text{ AI}}4{\text{SI}}_{4} {\text{O}}_{10} ({\text{OH}})_{2} \cdot {\text{nH}}_{2} {\text{O}}$$

n is the number of water molecules.

The stress collapse effect of rock also causes the high-strength material Si–O inside the rock to transform into other less strong materials. Such as:

$${-}{\text{Si}} {-} {\text{O}} {-} {\text{Si}} + {\text{H}}_{2} {\text{O}} \to {\text{ Si}} {-} {\text{OH}} + {-} {\text{OH}} {-} {\text{Si}} {-}$$

Adsorption: adsorption plays a pivotal role in mediating the interaction between rock and water. Initially, when water infiltrates the interior of the rock, it occupies a substantial volume within its matrix. Subsequently, under the influence of dynamic loading, the micro-pores and fissures within the rock gradually expand. This amplifies the adsorption effect, leading to heightened water adsorption on the surface of minerals present within the rock, such as kaolinite and montmorillonite. Consequently, this adsorption process engenders internal tension within the rock, consequently diminishing its overall strength.

The presence of capillary forces between water and rock particles remarkably affects the rock mechanical properties. When the rock becomes saturated with water, it absorbs a significant quantity of water into its internal structure, resulting in the expansion of rock particles and a subsequent reduction in capillary force. This decline in capillary force, in turn, results in a corresponding reduction in the strength of rock.

In light of the foregoing analysis, it becomes evident that the influence mechanism of water-dynamic load coupling on sandstone strength is chiefly governed by chemical reactions, adsorption, and the capillary forces engendered by the interaction between rock particles and water. However, it is worth noting that chemical reactions typically entail lengthy processes. Therefore, in laboratory testing, the primary mechanisms influencing the strength of sandstone due to water are predominantly adsorption and the capillary forces generated by the interaction between rock particles and water.

### Energy calculation principle and comprehensive evolution characteristics of energy

**Figure 7:**
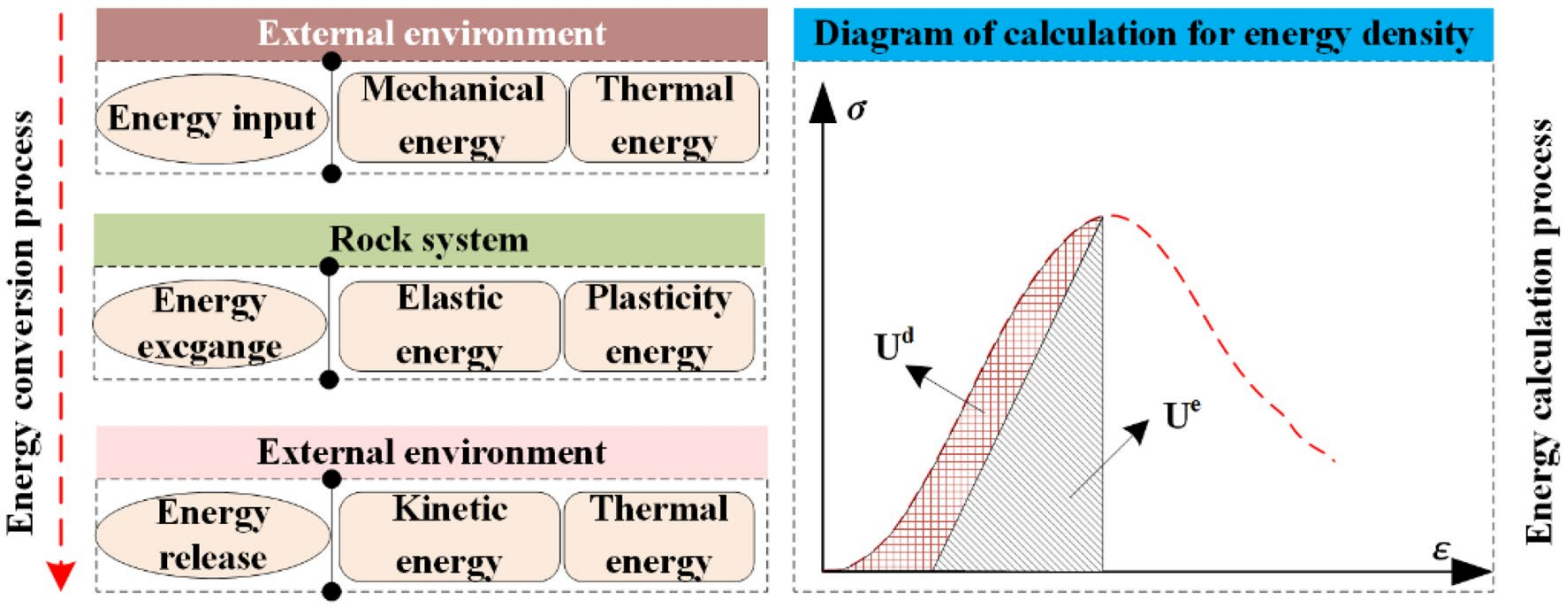
Schematic diagram of energy calculation principle.

Numerous studies have explored the incident energy, transmitted energy, and other energy conversions within rocks subjected to dynamic loading. Nonetheless, limited attention has been directed towards elucidating the energy conversion association between dissipated and elastic energy within rocks experiencing dynamic loads. It is crucial to acknowledge that the internal energy conversion mechanism within rocks can vary under different impact velocities and varying water conditions, as depicted in Fig. 7.

This research specifically centers on unraveling the internal energy conversion mechanism inherent to natural, dry, and saturated sandstone under diverse impact velocities. The use of unidirectional load during the dynamic load impact tests on saturated, dry, and natural sandstone at various impact speeds requires the computation of the total energy ‘U’ within the sandstone, employing the following formula^47–49^:

2 $$U = U^{{\text{e}}} + U^{{\text{d}}}$$

3 $${\text{where}}\quad \quad U = \int_{0}^{{\varepsilon_{1} }} {\sigma_{1} } d\varepsilon_{1}$$

4 $$U^{{\text{e}}} = \frac{1}{2}\sigma_{1} \varepsilon_{1}^{{\text{e}}} = \frac{1}{{2E_{{\text{u}}} }}\sigma_{1}^{2}$$

5 $$U_{{\text{d}}} { = }U{ - }U^{{\text{e}}}$$

In which, σ_1_ and ε_1_ denote the axial principal stress and strain, separately, and E_u_ represents the corresponding elastic modulus.

**Figure 8:**
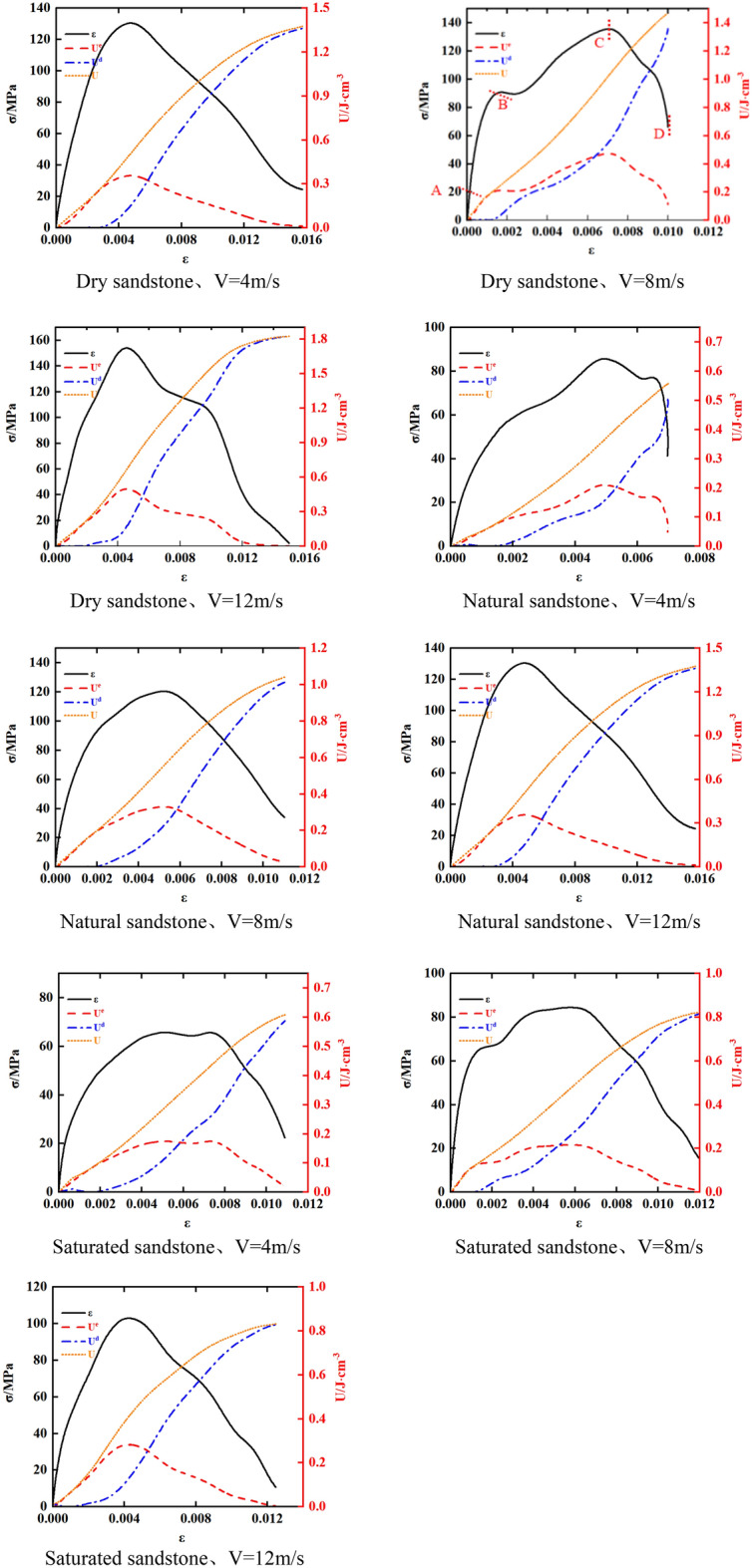
Energy evolution characteristics of sandstone.

Figure 8 elucidates the energy evolution process encompassing dry sandstone, natural sandstone, and saturated sandstone, spanning from the initial deformation to failure, across a spectrum of impact velocities. The energy curves corresponding to each sandstone type manifest four well-defined stages.

OA stage: the initial application of dynamic load results in an increase in elastic, dissipated, and total energy across dry sandstone, natural sandstone, and saturated sandstone subjected to various impact velocities. Notably, the rate of increase in elastic energy generally surpasses that of dissipated energy during this stage. This phenomenon can be attributed to the relatively brief duration of the sandstone compaction phase within this stage.

Within the compaction stage, the energy transferred from the external system to the rock samples undergoes a partial conversion into elastic energy, while the remainder is dissipated as heat. As the dynamic load intensifies, both the applied load on the rock and its corresponding deformation experience augmentation.

AB stage: as the dynamic load stress continues to increase, the elastic, dissipative as well as total energy of dry sandstone, natural sandstone, and saturated sandstone exhibit varying increments at different impact velocities. This behavior can be attributed to the occurrence of linear elastic deformation in sandstone under the effect of dynamic load. During this stage, the energy transferred from the external system to the rock samples is predominantly converted into elastic energy, with a smaller portion dissipated as heat. Furthermore, at this stage, the rock samples' deformation exhibits almost linear elastic behavior. Consequently, the elastic, dissipative as well as total energy of dry sandstone, natural sandstone, and saturated sandstone display increases corresponding to different impact velocities.

BC stage: with the sustained rise in dynamic load stress, the elastic, dissipative and total energies exhibited by dry sandstone, natural sandstone, and saturated sandstone demonstrate varying increments across different impact velocities. This behavior can be attributed to the onset of linear elastic deformation within sandstone induced by dynamic loading.

CD stage: the continued increase in dynamic load stress leads to an upward trend in the total and dissipated energies presented by dry, saturated and natural sandstone at various impact velocities, concurrently with a decrease in elastic energy. This distinctive behavior is primarily attributed to the prevalence of plastic deformation within the CD stage, where the majority of the energy stored in the sandstone undergoes conversion into dissipative energy. During this stage, the rock experiences plastic deformation, rendering elastic deformation negligible.

U^e^_A_, U^d^_A_ and U_A_ denote the elastic, dissipative and total energy corresponding to the critical point A at the compaction stage, U^e^_B_, U^d^_B_ and U_B_ denote the elastic, dissipative and total energy corresponding to the critical point B at the elastic stage, U^e^_C_, U^d^_C_ and U_C_ denote the elastic, dissipative and total energy corresponding to the critical point C at the damage stage, and U^e^_D_ and U_D_ denote the elastic and total energies corresponding to the critical point D at the post-peak stage, respectively.

**Figure 9:**
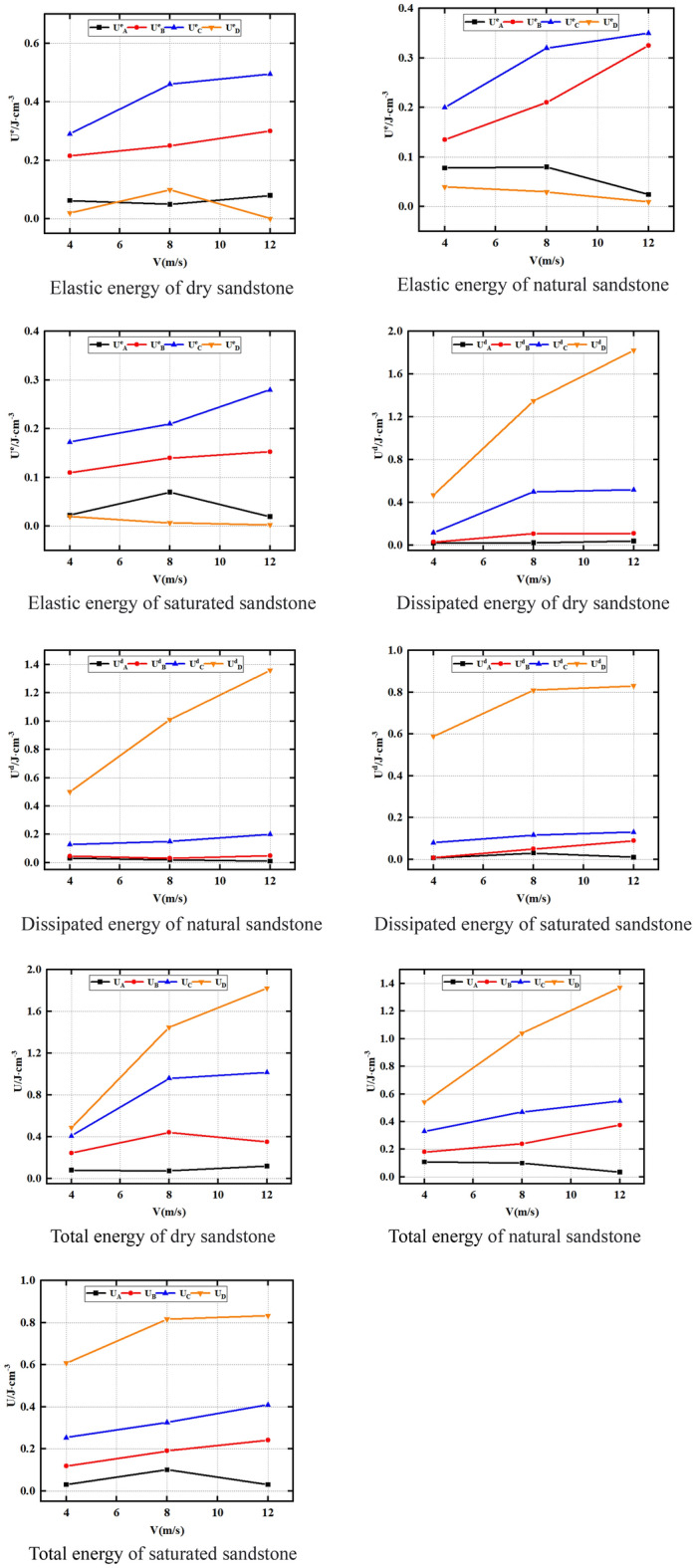
Energy characteristics of sandstone under various impact velocities.

The impact velocity and water saturation rate are significantly correlated with sandstone energy evolution state. In addition, the impact velocity and water saturation rate can significantly change the intrinsic properties of sandstone and thus its energy state. As can be seen from Fig. 9, under dry, saturated and natural conditions, U^e^_B_ and U^e^_C_ increase with the increase of impact velocity; however, there is no obvious rule for U^e^_A_ and U^e^_D_ as impact velocity raises. This indicates that the impact velocity significantly affects the U^e^_B_ and U^e^_C_ of sandstone, but has no significant effect on U^e^_A_ and U^e^_D_. This is owing to the rock loading rate is larger under dynamic load conditions, and its compaction stage is less obvious than the static load compaction stage. Therefore, the impact velocity has no obvious rule on U^e^_A_. In addition, U^e^_D_ represents the elastic energy existing inside the damaged and unstable rock, and after the damaged and unstable rock, the internal elastic energy is almost all transformed into dissipative energy, that is, the elastic energy is close to 0, which is also proved by our tests. For example, under dry conditions, the U^e^_D_ of sandstone under different impact velocities is 0.02, 0.04 and 0.02 J cm^−3^ respectively. Therefore, the impact velocity has no significant impact on the U^e^_D_ of sandstone. Simultaneously, this is because with the increase of impact speed, the load on the rock sample will increase, caused by the impact effect, the strength of the rock will increase, while its deformation degree will increase, so its energy will increase.

In addition, as can be seen from Fig. 9, U^d^_B_, U^d^_C_, U^d^_D_, U_B_, U_C_ and U_D_ almost all show a law of increasing as the impact velocity raises under dry, natural and saturated conditions; however, U^d^_A_ and U_A_ show no obvious law as the impact velocity raises. This indicates that the impact velocity significantly affects U^d^_B_, U^d^_C_ and U^d^_D_ of sandstone, but has no significant effect on U^d^_A_ and U_A_. This is owing to as the impact velocity raises, the energy absorbed by the sandstone increases, and the breakage degree of the sample increases, so the dissipative energy increases.

**Figure 10:**
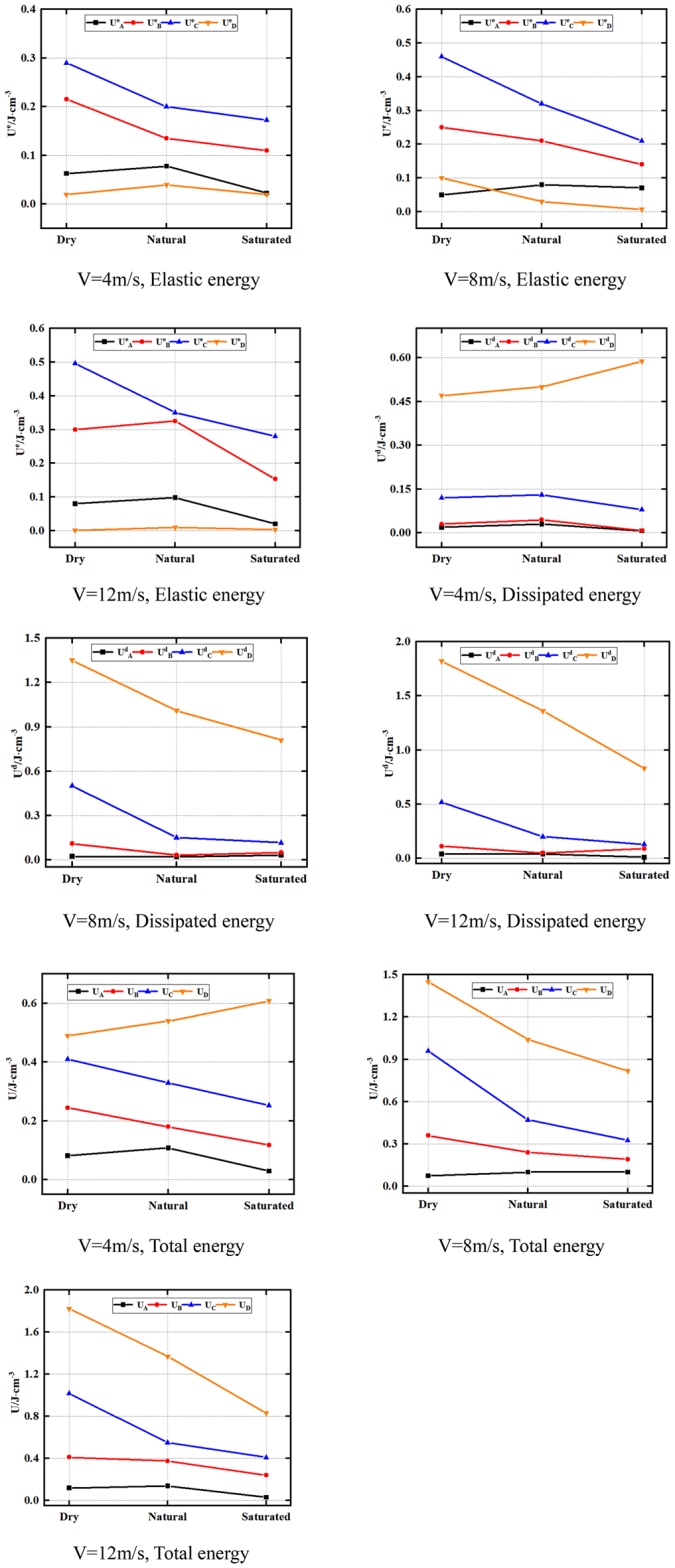
Energy properties of sandstone at varying contents of water.

In addition, when the impact velocity is the same, the water content of the sandstone will also affect its energy conversion law. Therefore, Fig. 10 shows U^e^_A_, U^e^_B_, U^e^_C_ and U^e^_D_ of natural, dry and saturated sandstone at the same impact velocity. U^d^_A_, U^d^_B_, U^d^_C_, U^d^_D_; U_A_, U_B_, U_C_, U_D_. As can be seen from Fig. 10, U^e^_B_ and U^e^_C_ of natural rock sample > U^e^_B_ and U^e^_C_ of dry rock sample > U^e^_B_ and U^e^_C_ of saturated rock sample under the same impact velocity. For instance, at 4 m/s impact velocity, the U^e^_B_ of natural rock sample, dry rock sample and saturated rock sample are 0.215, 0.135 and 0.11 J cm^−3^, respectively. The U^e^_C_ of natural rock sample, dry rock sample and saturated rock sample are 0.29, 0.2, and 0.173 J cm^−3^, respectively. The rock U^e^_D_ and U^e^_A_ are not significantly impacted by the moisture content. This is due to the fact that when the water content rises, both its strength and strain fall, so its internal energy decreases. In addition, as mentioned above, the compaction stage of sandstone is not obvious under dynamic load conditions. Therefore, the moisture condition has no significant effect on the U^e^_A_ and U^e^_D_ of the rock.

### Evolution law of ratio of elastic to dissipative energy

We determine the value of the ratio “s” between dissipated and elastic energy. Subsequently, we chart the strain evolution curve encompassing the progression from deformation to instability failure for natural, dry, and saturated rock samples subjected to different impact velocities.

**Figure 11:**
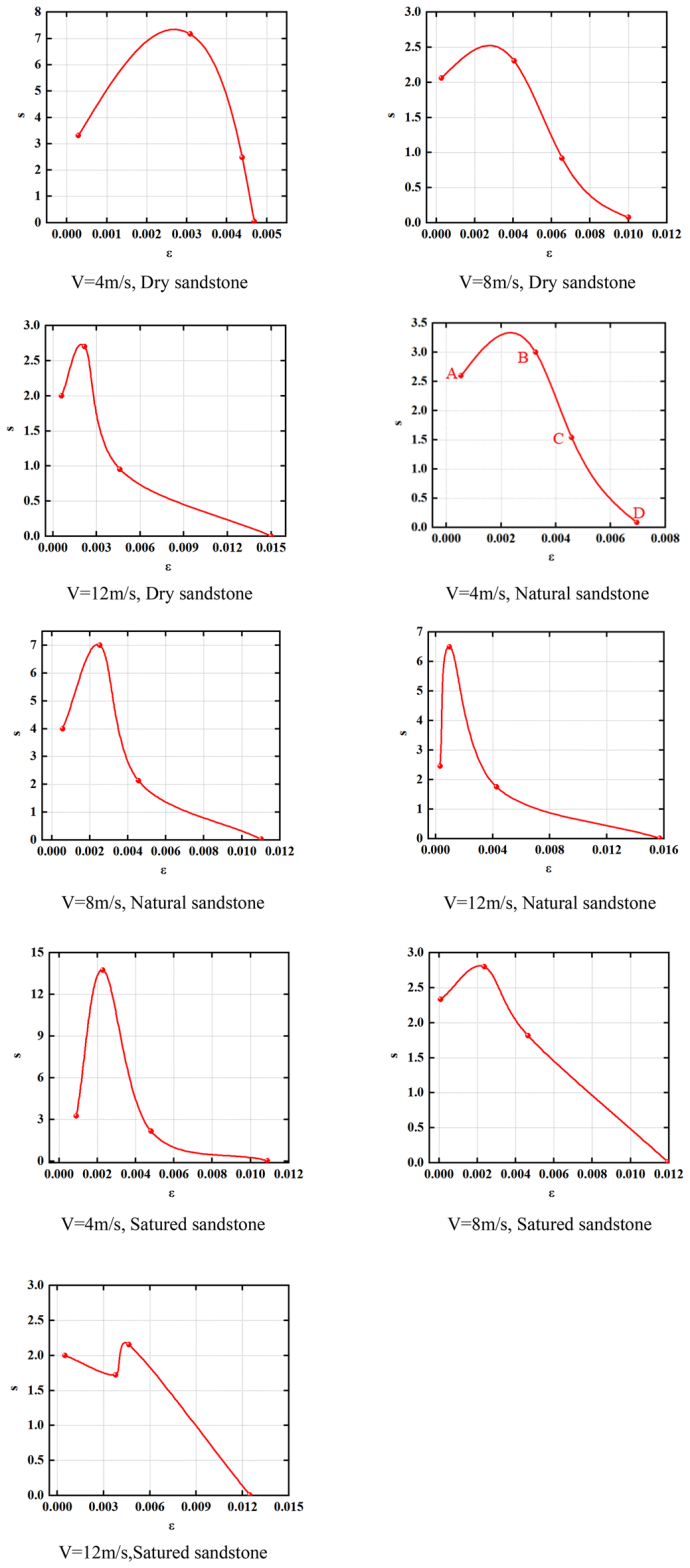
Energy characteristics of sandstone under different water content.

In Fig. 11, the ratio (s) of elastic energy to energy consumption in natural, dry, and saturated rock samples during the deformation process leading to instability failure at different impact velocities follows a distinctive pattern. As strain levels increase, the s value initially ascends and subsequently declines.

For example, with 4 m/s impact velocity at the end of the compaction step (point A in Fig. 11), the s value for natural sandstone measures 2.6. Advancing to the end of the elastic stage (point B in Fig. 11) at the same impact velocity, the s value for natural sandstone rises to 3. This elevation in the s value from point A to point B signifies distinct energy conversion mechanisms at different stages of the rock samples. During the compaction stage, where internal micro-cracks are compressed and sealed, the elastic energy slightly surpasses the dissipated energy. However, at the end of the elastic stage, the elastic energy significantly surpasses the dissipated energy due to most of the energy being channeled into elastic deformation. At the peak point (point C in Fig. 11) at 4 m/s impact velocity, the s value for natural sandstone measures 1.53. The transition from point B to C demonstrates a shift in the energy conversion mechanism within the sample of rock. At the conclusion of the elastic stage, fractures and damage appear while elastic energy predominates, leading to plastic deformation and a substantial increase in dissipated energy. Consequently, the s value decreases.

Progressing from point C to point D, which corresponds to point D in Fig. 11 with 4 m/s impact velocity, the s value for natural sandstone further decreases and approaches zero. This decrease is attributed to the occurrence of instability failure in the sandstone at point D. When the sandstone moves from point C to D, macro instability failure takes place within the rock sample, resulting in a significant surge in dissipated energy relative to elastic energy. Furthermore, from point C to point D, the sandstone reaches its peak strength, predominantly experiencing plastic deformation, with elastic energy primarily transforming into dissipated energy. Hence, the s value for the rock sample at point D approaches zero.

Additionally, maintaining a constant water content, the impact velocity does not modify the general pattern of the S-strain curve. Nevertheless, it does serve a remarkable effect on the specific S-values at each data point. Similarly, the S-values of natural, dry, and saturated sandstones exhibit variations even when subjected to the same impact velocities, but the overall trajectory of the S-strain curve remains uniform.

### Energy ratio

The time effect also affects the energy conversion law of rocks. Therefore, in the current work, the ratio of each energy of the rock to its corresponding time is defined as the energy ratio, as follows:

6 $$\left\{ \begin{gathered} {\text{K}}^{{\text{e}}} = \frac{{U^{{\text{e}}} }}{{\text{t}}} \hfill \\ {\text{K}}^{{\text{d}}} = \frac{{U^{{\text{d}}} }}{{\text{t}}} \hfill \\ {\text{K}} = \frac{U}{{\text{t}}} \hfill \\ \end{gathered} \right\}$$

K^e^_A_, K^d^_A_ and K_A_ denote the elastic, dissipative and total energy ratio corresponding to the critical point A at the compaction stage, respectively. K^e^_B_, K^d^_B_ and K_B_ denote the elastic, dissipative and total energy ratio corresponding to the critical point B at the elastic stage, respectively. K^e^_C_, K^d^_C_ and K_C_ denote the elastic, dissipative and total energy ratio corresponding to the critical point C at the damage stage, respectively. K^e^_D_, K^d^_D_ and K_D_ denote the elastic, dissipative and total energy ratio corresponding to the critical point D in the post-peak stage, respectively.

**Figure 12:**
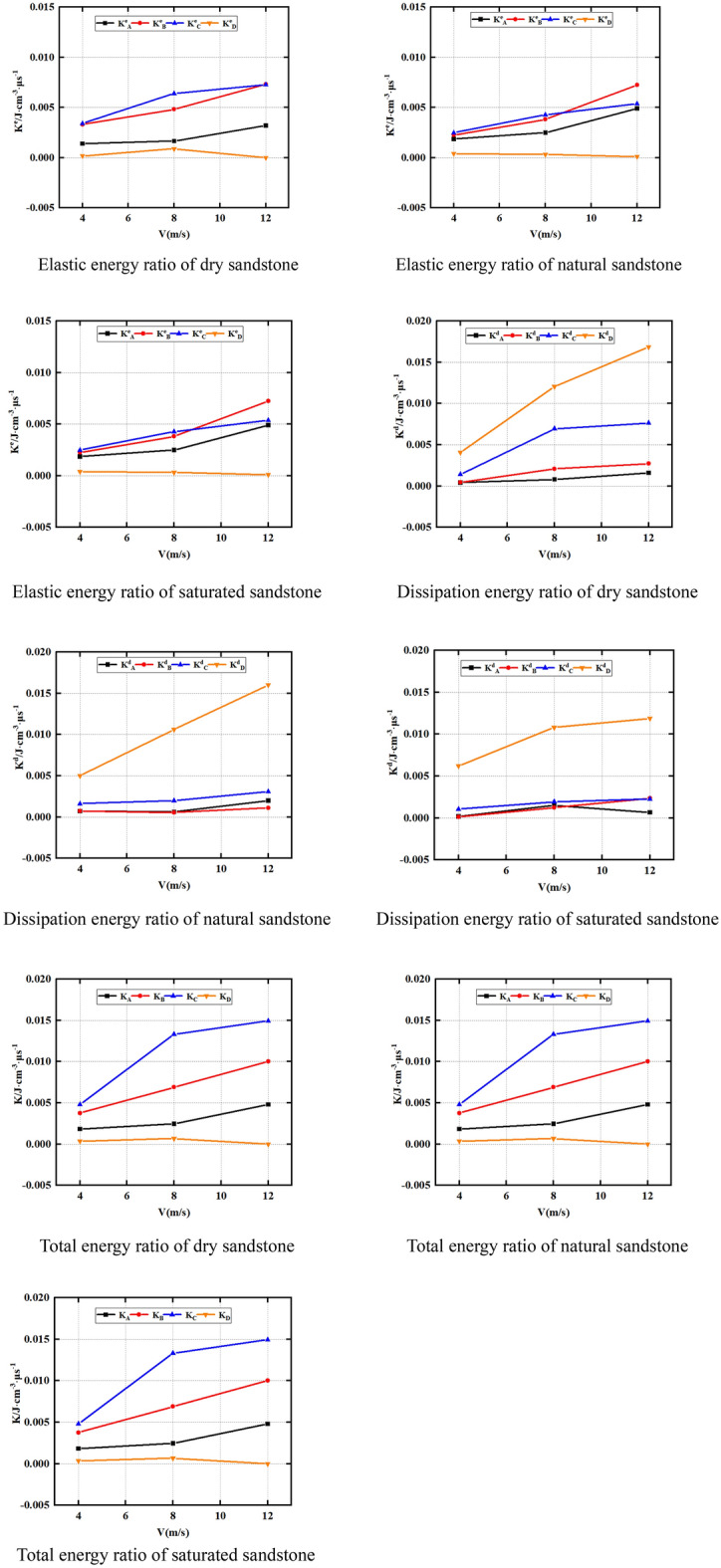
Evolution law of energy ratio of sandstone under various impact velocities.

Figure 12 presents the strong relationship between the sandstone energy ratio and the impact velocity and water saturation rate. Moreover, the impact velocity and water saturation rate can significantly change the intrinsic properties of sandstone and thus its energy state. K^d^_A_, K^d^_B_, K^d^_C_, K^d^_D_; K_A_, K_B_, K_C_, K_D_. Figure 12 displays that K^e^_A_, K^e^_C_ and K^e^_B_ increase as the impact velocity raises under dry, natural and saturated conditions, while K^e^_D_ has no significant relationship with impact velocity. This indicates that the impact velocity significantly affects the K^e^_A_, K^e^_C_ and K^e^_B_ of the rock sample, while the rock sample’s K^e^_D_ is not evidently impacted by the impact velocity. This is owing to when the sandstone is at the critical point D of the post-peak stage, the sandstone elastic energy is almost completely dissipated, so the impact velocity has no significant rule on the K^e^_D_ of the sandstone.

In addition, it can be seen from Fig. 12 that K^d^_B_, K^d^_C_, K^d^_D_, K_B_, K_C_ and K_D_ almost all show a law of increasing as the impact velocity elevates under dry, natural and saturated conditions. However, K^d^_A_ and K_A_ show no obvious law as the impact velocity elevates. This indicates that the impact velocity has significant effects on K^d^_B_, K^d^_C_ and K^d^_D_ of sandstone, but has no significant effects on K^d^_A_ and K_A_. This is owing to as the impact velocity elevates, the energy absorbed by the sandstone increases, and the degree of breakage of the sample increases, so the dissipative energy increases. In addition, as the impact velocity raises, the rock fracture time decreases. Therefore, the sandstone’s energy ratio increases with the increase of the impact velocity.

**Figure 13:**
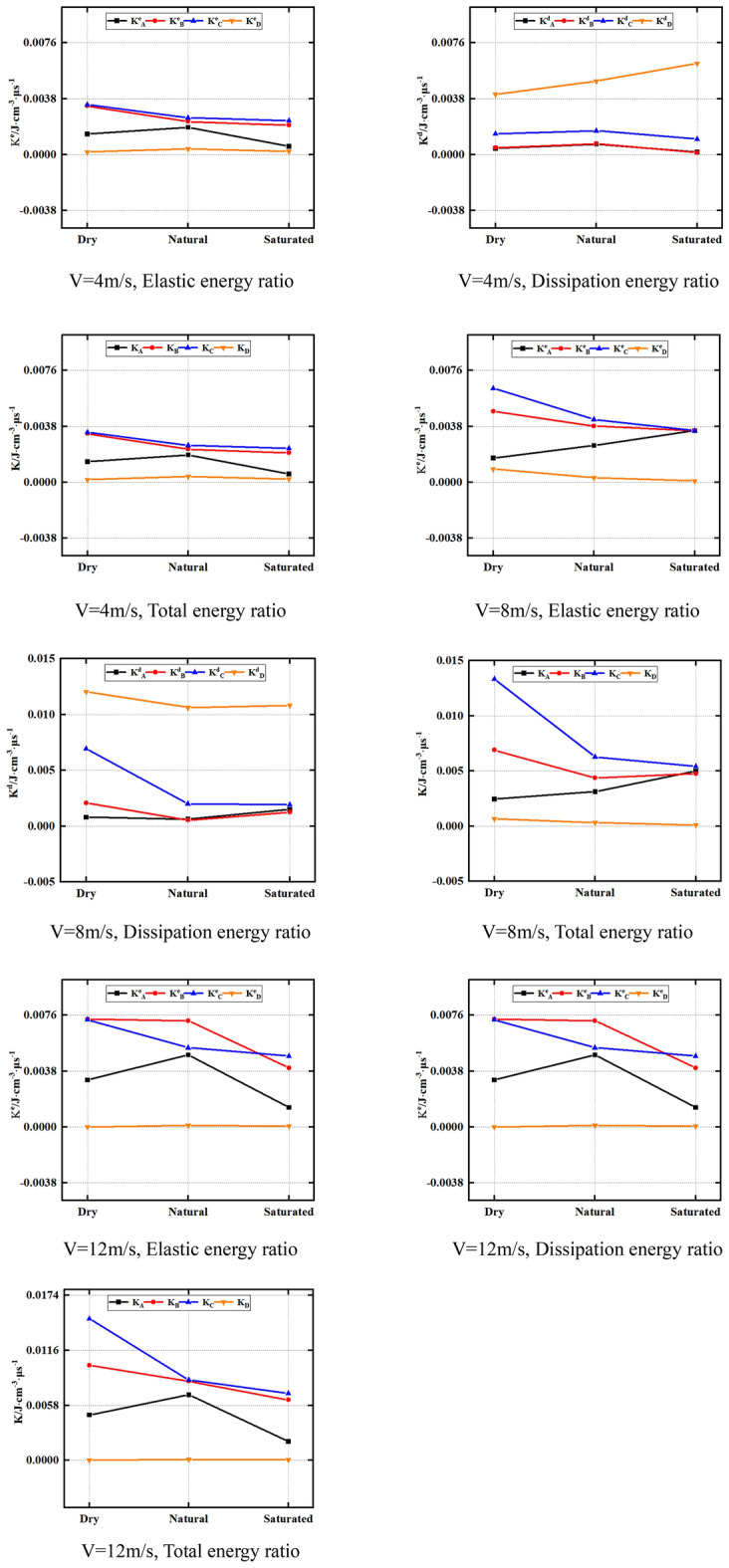
Evolution law of energy ratio of sandstone under different water content.

Furthermore, it’s essential to acknowledge that, when the impact velocity remains constant, the sandstone water content will also influence its energy ratio. The water content of sandstone also influences its energy ratio. In this regard, Fig. 13 provides insights into K^e^_A_, K^e^_B_, K^e^_C_ and K^e^_D_ of natural, dry and saturated sandstone under identical impact velocities. K^d^_A_, K^d^_B_, K^d^_C_, K^d^_D_; K_A_, K_B_, U_C_, K_D_. Figure 13 exhibited that K^e^_B_ and K^e^_C_ of natural rock sample > K^e^_B_ and K^e^_C_ of dry rock sample > K^e^_B_ and K^e^_C_ of saturated rock sample under identical impact velocities. The moisture condition has no remarkable influence on the K^e^_D_ and K^e^_A_ of rocks. Indeed, the decrease in strength and strain with raising content of water leads to a corresponding reduction in the internal energy of sandstone. Furthermore, as highlighted previously, the compaction stage of sandstone is less pronounced under dynamic load conditions. As a result, the moisture condition exhibits minimal influence on the K^e^_A_ and K^e^_D_ values of rocks.

Furthermore, Fig. 13 reveals that under 4 m impact velocity, K^d^_D_ and K_D_ of natural rock sample are less than K^d^_D_ of dry rock sample and K_D_ is less than K^d^_D_ and K_D_ of saturated rock sample. However, under the impact velocity of 4 m and 12 m, U^d^_D_ of natural rock sample, U_D_ > U^d^_D_ of dry rock sample, U_D_ > U^d^_D_ and U_D_ of saturated rock sample. Moreover, U^e^_B_ and U^e^_C_ of natural rock sample > U^e^_B_ and U^e^_C_ of dry rock sample > U^e^_B_ and U^e^_C_ of saturated rock sample.

### Microscopic rupture mechanism

**Figure 14:**
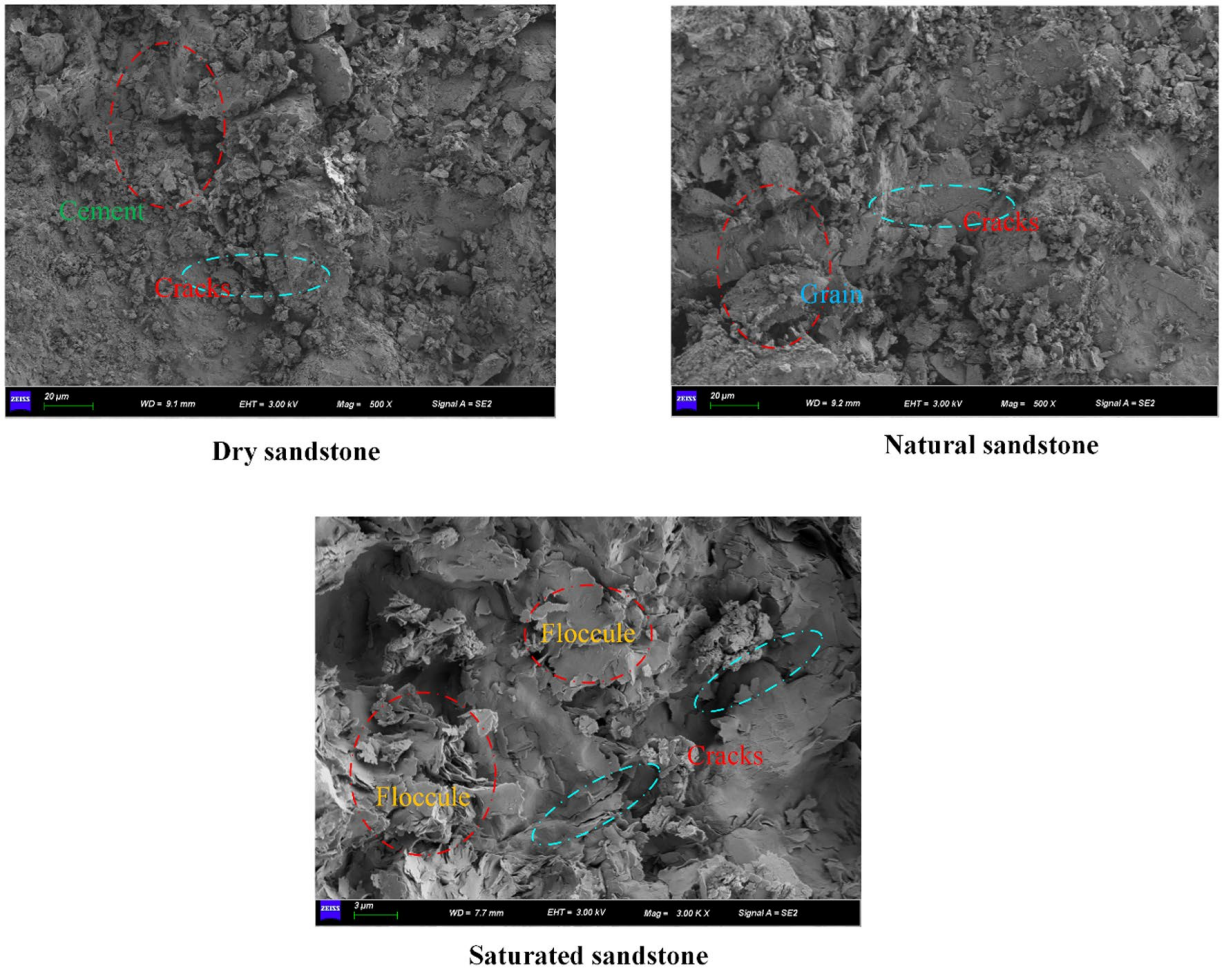
SEM image of sandstone under different water content.

**Figure 15:**
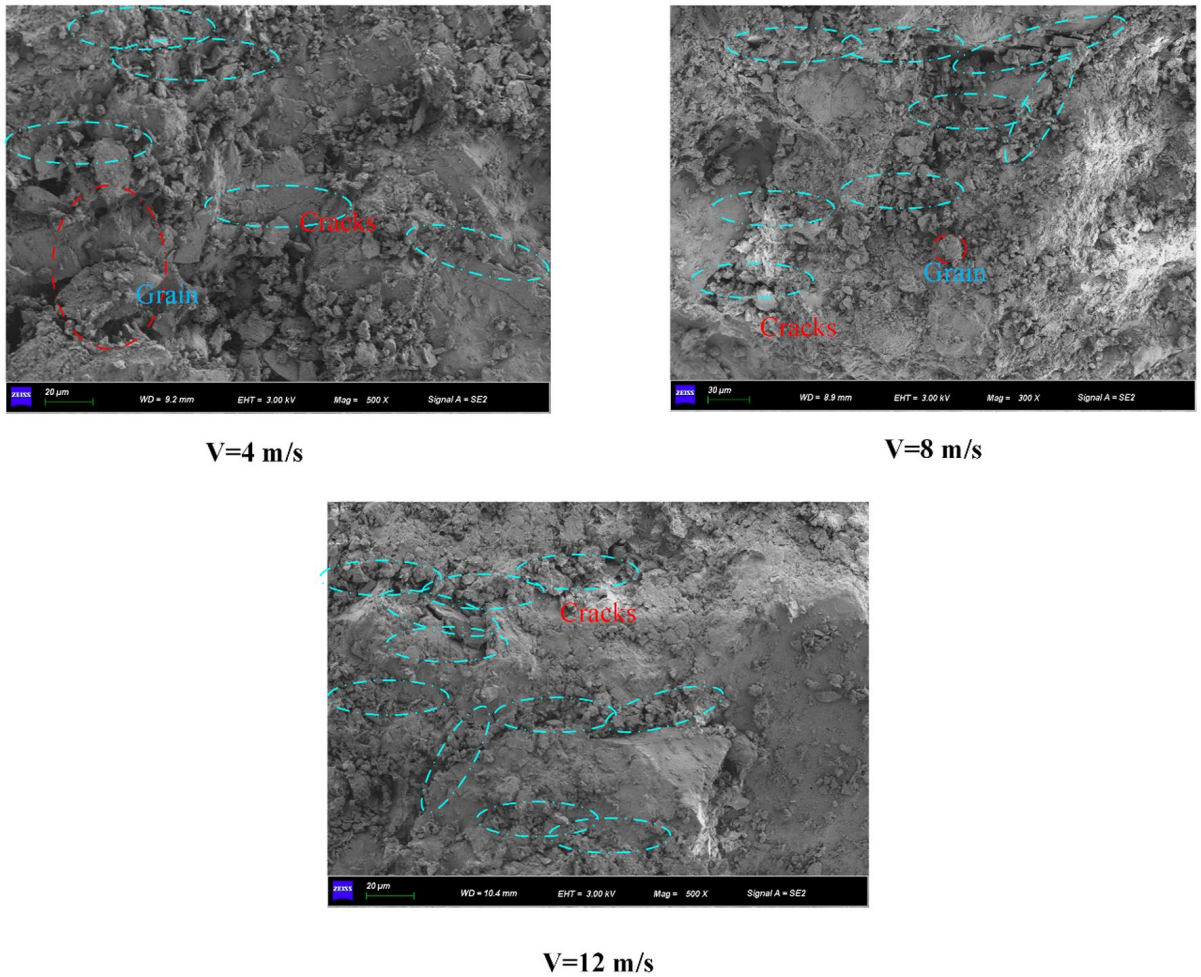
SEM image of sandstone under different impact velocities.

It is an important part of the study of rock instability failure to explore the micro-fracture mechanism of rock^44,50,51^. Thus, it is imperative to examine the sandstone micro-fracture process at diverse impact velocities in saturated, natural, and dry environments. This study examines the microscopic fracture mechanism of sandstone through SEM scanning tests. As depicted in Fig. 14, the microscopic images of dry sandstone reveal evident cracks and defects within the rock samples at the same impact velocity. Additionally, the microscopic particle cementation of dry sandstone appears notably dense, indicative of robust mechanical properties. In contrast, the microscopic image of natural sandstone displays alterations in the microstructure of the rock samples when compared to dry sandstone. The presence of water within natural sandstone results in the enlargement of microscopic particles due to moisture absorption, leading to dissolution, aggregation, and the formation of elliptical and circular-shaped flocs, accompanied by localized small voids. Conversely, the microscopic image of saturated sandstone portrays a more pronounced deterioration in interparticle cementation due to the infiltration of a significant volume of water. The heightened particle fullness and flocculent arrangement of particles contribute to the extensive development of initial micro-cracks, resulting in a looser structure. This transformation is particularly prominent in argillaceous siltstone, a cemented rock, where the existence of water induces substantial varies in the mechanical behavior of sandstone. Furthermore, as illustrated in Fig. 15, the impact velocity exerts a profound influence on the sandstone’s micro-fracture mechanism, with higher impact velocities correlating as the number of cracks on the micro-fracture surface elevated. This observation indicates that the degree of damage in sandstone escalates with higher impact velocities, as the micro-cracks within the sandstone section become more pronounced.

## Discussion

Previous studies have extensively researched the sandstone mechanical behavior under a variety of moisture contents and coupled dynamic loads, but investigations into the sandstone’s internal energy conversion mechanisms under these conditions have been lacking. Dynamic impact tests were performed in this work on dry, natural, and saturated sandstones at diverse impact velocities, delving into the internal energy conversion mechanisms of sandstone. The research findings hold significant importance for rock engineering. As described in this study, as the content of moisture elevates, the accumulated energy within the sandstone decreases. This implies that in rock engineering, if appropriate saturation can be achieved, the energy stored within the rock diminishes, making the rock more prone to unstable failure. Furthermore, under the same moisture content, different dynamic loads correspond to different energy conversion mechanisms in the rock. Therefore, in rock blasting engineering, it is crucial to finely tune the magnitude of dynamic loads to quantify the sandstone’s fragmentation level and energy conversion. Moreover, although this study investigated the sandstone internal energy evolution mechanisms under diverse contents of moisture and dynamic loads, the gradual fracture mechanisms during the fracturing process remain unknown. Based on this, in future research, we can employ CT scanning methods to investigate the gradual fracture mechanisms of sandstone under these conditions.

## Conclusion

The research findings have revealed that moisture content and dynamic loading significantly influence the sandstone internal energy conversion mechanisms. These findings might be extremely valuable in understanding how sandstone dissipates energy and fails under diverse dynamic stress scenarios and moisture contents.

1. The impact velocity is proportional to the strength of rock, and dry sandstone is stronger than natural sandstone and higher than saturated sandstone.
2. The energy evolution of rock samples can be categorized into various stages from initial deformation to instability failure under the dynamic load-water coupling. The law of energy evolution is different in different stages.
3. A new coefficient energy ratio considering time is proposed. As the impact velocity raises, K^e^_A_, K^e^_C_, K^e^_B_, K^d^_B_, K^d^_C_, K^d^_D_, K_B_, K_C_ and K_D_ almost all show the law of increasing as the impact velocity raises.
4. With the change of rock sample from dry to saturated, the cementation of dry sandstone particles changes from dense to loose.

While we have implemented study on the internal energy evolution mechanisms of sandstone under different moisture contents and dynamic loading conditions, the gradual fracture mechanisms during the fracturing process remain unknown. Based on this, in future research, we can utilize CT scanning methods to investigate the gradual fracture mechanisms of sandstone under these conditions.

## Data Availability

Upon reasonable request, the corresponding author will provide the data supporting the study conclusions.
